# Clinical utility of circulating tumor DNA for molecular assessment in pancreatic cancer

**DOI:** 10.1038/srep18425

**Published:** 2015-12-16

**Authors:** Erina Takai, Yasushi Totoki, Hiromi Nakamura, Chigusa Morizane, Satoshi Nara, Natsuko Hama, Masami Suzuki, Eisaku Furukawa, Mamoru Kato, Hideyuki Hayashi, Takashi Kohno, Hideki Ueno, Kazuaki Shimada, Takuji Okusaka, Hitoshi Nakagama, Tatsuhiro Shibata, Shinichi Yachida

**Affiliations:** 1 Division of Cancer Genomics, National Cancer Center Research Institute, Tokyo 1040045, Japan; 2 Department of Hepatobiliary and Pancreatic Oncology, National Cancer Center Hospital, Tokyo 1040045, Japan; 3 Hepatobiliary and Pancreatic Surgery Division, National Cancer Center Hospital, Tokyo 1040045, Japan; 4 Department of Bioinformatics, National Cancer Center Research Institute, Tokyo 1040045, Japan; 5 Division of Genome Biology, National Cancer Center Research Institute, Tokyo 1040045, Japan; 6 Division of Carcinogenesis and Cancer Prevention, National Cancer Center Research Institute, Tokyo 1040045, Japan; 7 Laboratory of Molecular Medicine, Human Genome Center, The Institute of Medical Science, The University of Tokyo, Tokyo 1088639, Japan

## Abstract

Pancreatic ductal adenocarcinoma (PDAC) remains one of the most lethal malignancies. The genomic landscape of the PDAC genome features four frequently mutated genes (*KRAS*, *CDKN2A*, *TP53*, and *SMAD4*) and dozens of candidate driver genes altered at low frequency, including potential clinical targets. Circulating cell-free DNA (cfDNA) is a promising resource to detect and monitor molecular characteristics of tumors. In the present study, we determined the mutational status of *KRAS* in plasma cfDNA using multiplex picoliter-droplet digital PCR in 259 patients with PDAC. We constructed a novel modified SureSelect-KAPA-Illumina platform and an original panel of 60 genes. We then performed targeted deep sequencing of cfDNA and matched germline DNA samples in 48 patients who had ≥1% mutant allele frequencies of *KRAS* in plasma cfDNA. Importantly, potentially targetable somatic mutations were identified in 14 of 48 patients (29.2%) examined by targeted deep sequencing of cfDNA. We also analyzed somatic copy number alterations based on the targeted sequencing data using our in-house algorithm, and potentially targetable amplifications were detected. Assessment of mutations and copy number alterations in plasma cfDNA may provide a prognostic and diagnostic tool to assist decisions regarding optimal therapeutic strategies for PDAC patients.

Pancreatic ductal adenocarcinoma (PDAC) is a devastating disease and the seventh leading cause of cancer death in the world^1^. Despite a rising incidence worldwide, tools for early detection remain unavailable and the prognosis is poor, with a 5-year survival rate limited to approximately 4–7% (http://seer.cancer.gov/statfacts/html/pancreas.html). To date, surgery is the only curative treatment for PDAC, but only 15 to 20% of patients present with localized, non-metastatic disease. The rapid organ metastasis observed in some patients treated with potentially curative surgery suggests that occult distant metastases may happen very early, negating survival benefit in the subset of operable patients.

Whole-genome or exome sequencing analyses have revealed that somatic alterations of the PDAC genome often occur in four genes, namely *KRAS*, *CDKN2A*, *TP53* and *SMAD4*^2^,^3^,^4^, and at low frequency in dozens of candidate driver genes. Jones and colleagues have determined driver genes in PDAC that are associated with U.S. Food and Drug Administration (FDA)-approved therapies (www.fda.gov/Drugs/) for oncologic indications or therapies in published prospective clinical studies^5^. They identified somatic alterations in genes with target potential in approximately 20% of PDAC patients. Precision medicine strategies using genomic profiling are particularly pertinent for PDAC. Although tumor tissue is the gold standard for sequencing, major barriers exist in terms of its acquisition. Studies for molecular screening often exclude some patients because of inability to obtain a biopsy, insufficient or no tumor content in the available specimen, or deteriorating performance status^6^,^7^. Most importantly, biopsies are not without clinical complications^8^.

Circulating cell-free DNA (cfDNA) exists as small DNA fragments in blood. The presence of tumor-derived DNA in cfDNA, also known as circulating tumor DNA (ctDNA), provides a less-invasive approach to diagnose cancers, monitor chemotherapy-resistant mutations and overcome the problem of tumor heterogeneity^9^,^10^,^11^, based on the concept of “liquid biopsy”. While detection of ctDNA is still challenging, since only a very small fraction of total cfDNA is derived from tumors^9^, advances in technology, including allele-specific PCR, BEAMing (beads, emulsion, amplification, magnetics)^12^, droplet digital PCR (ddPCR)^13^ and next-generation sequencing (NGS), are making it more feasible. Especially, picoliter-droplet digital PCR technologies (RainDance Technologies, Billerica, MA) possess high sensitivity and allow multiplex assays^14^,^15^. A NGS approach is more suitable to detect a wide range of cancer-related gene mutations than ddPCR. While previous studies demonstrated that ctDNA is detectable in a high proportion of patients with metastatic disease^16^, the clinical utility of ctDNA as a cancer biomarker is just beginning to be evaluated^17^. Though several studies have investigated ctDNA in PDAC patients^18^,^19^, further studies with a large sample size are required to establish the clinical utility of liquid biopsy in PDAC. Because mutations in *KRAS* are among the first to occur during carcinogenesis and they are observed in >90% of PDAC, we first evaluated *KRAS* mutations in plasma as a benchmark of ctDNA from 259 patients with PDAC, then analyzed correlations with clinicopathologic features. In addition, we performed targeted deep sequencing of cfDNA using the SureSelect-KAPA-Illumina platform to detect potentially therapeutically targetable mutations and copy number alterations in PDAC patients.

## Results

### Detection of *KRAS* mutations in cfDNA by ddPCR

**Figure 1:**
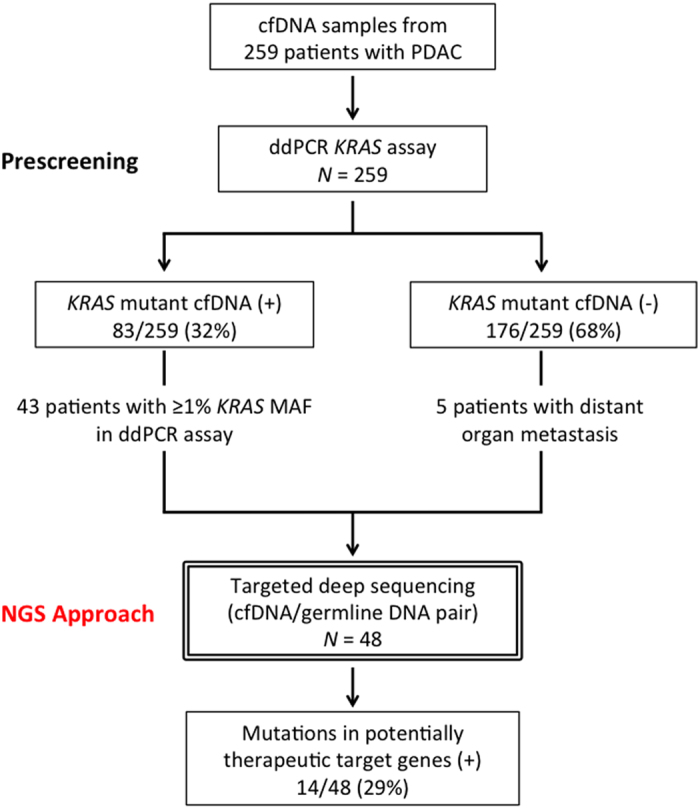
Patient flow diagram. MAF, mutant allele frequency.

**Figure 2:**
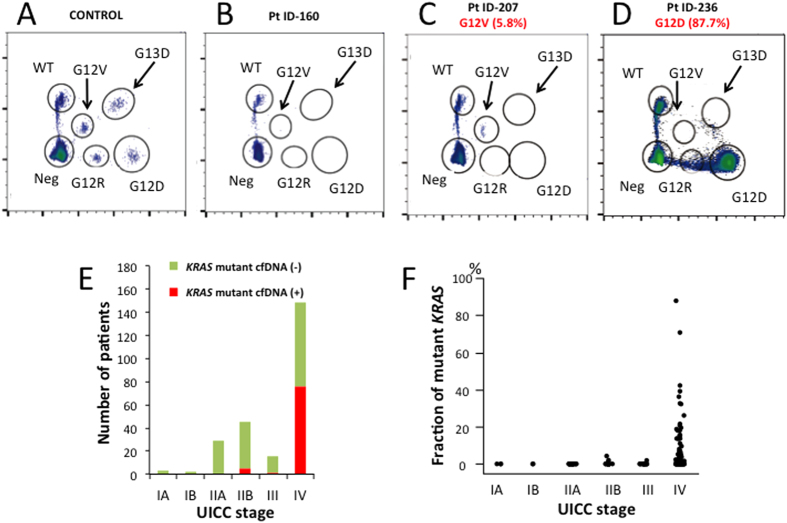
Two-dimensional histogram of the *KRAS* 5-plex assay for detection of *KRAS* mutations in plasma and relationship between detectability of mutant *KRAS* in plasma cell-free DNA (cfDNA) and UICC-stage. (**A**) Controls. Fragmented *KRAS* mutant and non-mutant genomic DNA reference standards were used. Representative views of droplet digital PCR plots using plasma cell-free DNA show no mutant *KRAS* alleles (**B**), G12V-mutant *KRAS* alleles (**C**) and G12D-mutant *KRAS* alleles (**D**). The *x*-axis and the *y*-axis correspond respectively to the FAM and VIC intensity (arbitrary units). (**E**) The number of patients with or without detectable levels of *KRAS* mutations in plasma in UICC-stages IA to IV. (**F**) The fraction of mutant *KRAS* in plasma cfDNA in UICC-stages IA to IV.

Two hundred and fifty-nine PDAC patients were involved in this study. Figure 1 shows the patient flow diagram. Clinicopathologic features are described in Supplementary Table S1. DNA was extracted from 2 mL plasma samples and quantified by a qPCR-based method (LINE-assay) in order to assess the amount of amplifiable cfDNA^20^. The median cfDNA concentration in plasma was 20.13 ng/2 mL plasma. Circulating cfDNA concentrations were significantly higher in UICC-stage IV cases when compared to UICC-stage I-III cases (median concentration: 21.65 ng/2 mL plasma vs. 17.59 ng/2 mL plasma, respectively, *P* = 0.0129) (Supplementary Figure S1A). However, cfDNA concentrations were not associated with the primary tumor size (Supplementary Figure S1B). It is known that >90% of PDAC harbor mutations in the *KRAS* gene as founder mutations^2^,^3^,^4^. Therefore, the presence of mutant *KRAS* DNA fragments in plasma from PDAC patients indicates the presence of ctDNA. A multiplex ddPCR assay was created to detect the four common *KRAS* mutations (G12D, G12V, G12R and G13D) (Supplementary Table S2), which account for 90% of all mutations of *KRAS* in PDAC (Fig. 2)^21^,^22^. In cases with ≥50% *KRAS* mutant allele frequency, amplification of the *KRAS* gene was suspected. Among 151 inoperable patients, mutant *KRAS* was detected in 63 of 107 (58.9%) PDAC patients with distant organ metastasis, while only 8 of 44 (18.2%) patients without distant organ metastasis showed detectable levels of mutant *KRAS* (Supplementary Figure S2). The presence of ctDNA was significantly (*P* < 0.0001) associated with the presence of distant organ metastasis.

In resectable PDAC patients, 9 of 108 (8.3%) had detectable levels of *KRAS* mutation in plasma cfDNA. Among them, five patients relapsed with metastatic tumors within six months after surgery, and had a very poor prognosis. A representative case is shown in Supplementary Figure S3. This patient was diagnosed with a 19 mm localized tumor with cystic lesions and no distant organ metastasis based on radiographic findings at the operation. A metastatic tumor in the liver was found by first follow-up CT three months after resection of the primary carcinoma, and the patient died after nine months. We evaluated the plasma sample obtained before surgery and detected 4.3% *KRAS* G12V. These findings suggested that detecting ctDNA might be useful to monitor tiny distant metastases that are hard to detect by routine medical imaging, which is in accordance with other recent findings^19^. Therefore, patients with borderline resectable PDAC and mutated *KRAS* gene detected in plasma might be spared the risk of surgery and have the benefit of preoperative chemotherapy.

We also analyzed the implications of ctDNA for clinical outcome. The results of univariate analysis using log-rank tests for the detection of mutated *KRAS* gene in plasma, as well as the clinical characteristics in relation to overall survival, are shown in Supplementary Table S3. The presence of mutant *KRAS* in plasma (*P* < 0.0001) (Supplementary Figure S4), as well as UICC-stage (stage I-III vs. stage IV, *P* < 0.0001), N-factor (*P* < 0.0001) or M-factor (*P* < 0.0001) in the TNM classification were significantly associated with poor overall survival. Multivariate analysis using the Cox proportional hazards model was conducted with the parameters that were significant at *P* < 0.05 in univariate analysis. As shown in Supplementary Table S4, multivariate analysis demonstrated detection of mutated *KRAS* gene in plasma by ddPCR (hazard ratio 3.04, *P* < 0.0001) and UICC-stage IV (vs. stage I-III, hazard ratio 2.05, *P* = 0.0153) to be independent prognostic factors for overall survival.

### Targeted deep sequencing of cfDNA with the Illumina platform

**Figure 3:**
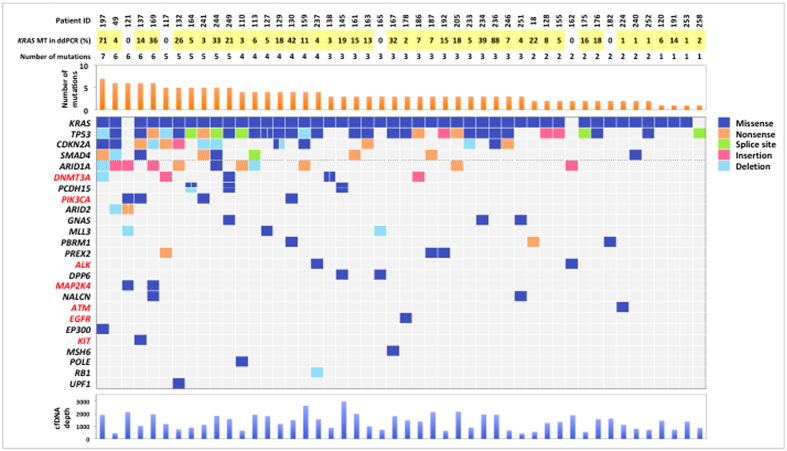
Somatic mutations detected by targeted sequencing of plasma cfDNA. The top bar plot shows the number of somatic mutations among 60 genes for tumors from each patient. The mutated genes detected by targeted deep sequencing are shown in the left-most column (arranged in descending order of number of mutations). Gene symbols of potential target genes are shown in *red*. The bottom bar plot shows the coverage depth for each cfDNA sample. MT, mutation.

We next performed targeted deep sequencing to further analyze mutated genes in cfDNA from PDAC patients using the Illumina platform (Illumina, Inc., San Diego, CA), which has the lowest error rate among high-throughput sequencing instruments^23^. To apply the SureSelect Target Enrichment System (Agilent Technologies, Santa Clara, CA) for Illumina paired-end sequencing of cfDNA, we optimized the library preparation conditions so that we could successfully reduce DNA sample input from 200 ng (the minimum input according to the manufacturer’s protocol) to “10 ng” by modifying SureSelect libraries for target enrichment through combination with the KAPA Hyper Prep Kit (Kapa Biosystems, Wilmington, MA). This workflow enabled targeted deep sequencing without increasing PCR duplicates in NGS. To our knowledge, this is the first report showing that the SureSelect Target Enrichment System with the KAPA Hyper Prep Kit can be applied for Illumina paired-end sequencing of plasma cfDNA. The sensitivity of detection of somatically mutated genes in plasma appeared to be dependent on the amount of cfDNA, the allele frequency of the mutations in the samples and the sequencing coverage. Therefore, we focused on samples with a relatively high (≥1%) *KRAS* mutant allele frequency in plasma cfDNA. Based on the results of *KRAS* multiplex ddPCR, 43 samples probably having substantial levels of ctDNA were selected for targeted deep sequencing. In addition, five cases were included even though they were negative for *KRAS* mutation by ddPCR, since they had distant organ metastases (Fig. 1). In order to achieve higher sequence coverage (~1,000x on average) cost-effectively and to test clinical feasibility, we designed an original gene panel for PDAC, focusing on 60 genes, including potential therapeutic targets (Supplementary Table S5). All 96 DNA libraries generated from 48 cfDNA and 48 matched germline DNA samples were pooled into one Flow Cell Illumina HiSeq2000. The unique coverage depth for cfDNA samples was 1,356x on average (range 417–2,955x) after excluding PCR duplication, and that for germline DNA samples was 1,492x on average (range 948–2,299x). Mutations detected by targeted deep sequencing are presented in Fig. 3 and Supplementary Table S6. All 48 patients had at least one mutation (median 3 mutations/patient, range 1–7 mutations).

*KRAS* mutation status was first evaluated as the benchmark with the Illumina platform. Examples were detected in 45 of 48 samples. In 42 of these, identical *KRAS* mutations were detected with both ddPCR and targeted sequencing (Supplementary Table S7). Comparing allele frequencies of mutated *KRAS* between ddPCR and targeted sequencing, there was a high degree of concordance (correlation coefficient, *r* = 0.94, *P* < 0.0001) (Supplementary Figure S5). Targeted sequencing identified a *KRAS* G12C mutation in one patient (Patient ID-182); this pattern (G12C) was not included in our multiplex ddPCR assay (G12D, G12R, G12V and G13D). In Patients ID-117 and ID-165, *KRAS* mutations were detected by targeted sequencing at mutant allele frequencies of 21.30% and 6.37%, respectively, although the ddPCR assay failed to detect these mutations. Conversely, the targeted sequencing could not detect *KRAS* mutation in Patient ID-258, with a 2.44% mutant allele frequency on ddPCR. Though we re-examined the sequencing data and found five mutation reads in this case (Patient ID-258), we considered this sample as negative for *KRAS* mutation due to low coverage depth (761x) in the region. No *KRAS* mutations were detected in the remaining two patients (Patient ID-121 and ID-162) by both ddPCR and NGS.

Importantly, mutations in potential therapeutic target genes were detected in 14 of 48 (29.2%) patients. These included *ALK* (n = 2), *ATM* (n = 1), *DNMT3A* (n = 5), *EGFR* (n = 1), *KIT* (n = 1), *MAP2K4* (n = 2), and *PIK3CA* (n = 4). In particular, three of four *PIK3CA* mutations (p.H1047L, p.E545K and p.Q546K) and one *EGFR* mutation (p.G724S) are known to be oncogenic and are *in vitro* therapeutic targets^24^,^25^,^26^.

### Estimation of targeted sequencing data by Ion AmpliSeq technology

Circulating cfDNA samples were available from 6 of 48 patients to confirm mutations in plasma using an alternative NGS technology, amplicon-based sequencing. We selected appropriate primers to obtain optimal amplicons for mutations detected in these six patients and carried out targeted sequencing with Ion AmpliSeq technology (Thermo Fisher Scientific, Waltham, MA) using cfDNA and matched germline DNA in six patients. The unique coverage depth for cfDNA samples was 2,352x on average (range 1,158–3,950x), and that for germline DNA samples was 23,231x on average (range 17,170–33,016x). The amplicon DNA sequencing identified the same alterations in 19/25 (76%) mutations detected by the SureSelect-KAPA-Illumina platform (Supplementary Table S8). Targeted resequencing by Ion AmpliSeq technology tended not to detect mutations, especially deletions, in regions with low sequencing depth.

### Validation of targeted sequencing data by whole-exome sequencing of a primary tumor sample

In order to examine how mutations in the primary carcinoma might be reflected in plasma cfDNA, we performed whole-exome sequencing using frozen tissue samples of the primary carcinoma of one patient (Patient ID-18). The average sequence depth of coding gene regions for the tumor sample was 189x and that for the germline DNA sample was 153x, which achieved >20-fold coverage of 96% of coding gene regions. We identified a total of 61 somatic non-synonymous mutations including 52 missense, 5 nonsense, and 1 splice-site mutations, as well as 3 deletions (Supplementary Table S9). Among 60 genes selected for targeted sequencing of cfDNA, mutations of two genes (*KRAS* and *PBRM1*) were detected by whole-exome sequencing, which exactly matched the mutational data from targeted deep sequencing of plasma cfDNA. We also confirmed the same point mutations in *KRAS* and *PBRM1* in the DNA of the primary carcinoma by Sanger sequencing (Supplementary Figure S6).

### Evaluation of somatic copy number alterations by targeted sequencing data of ctDNA

**Figure 4:**
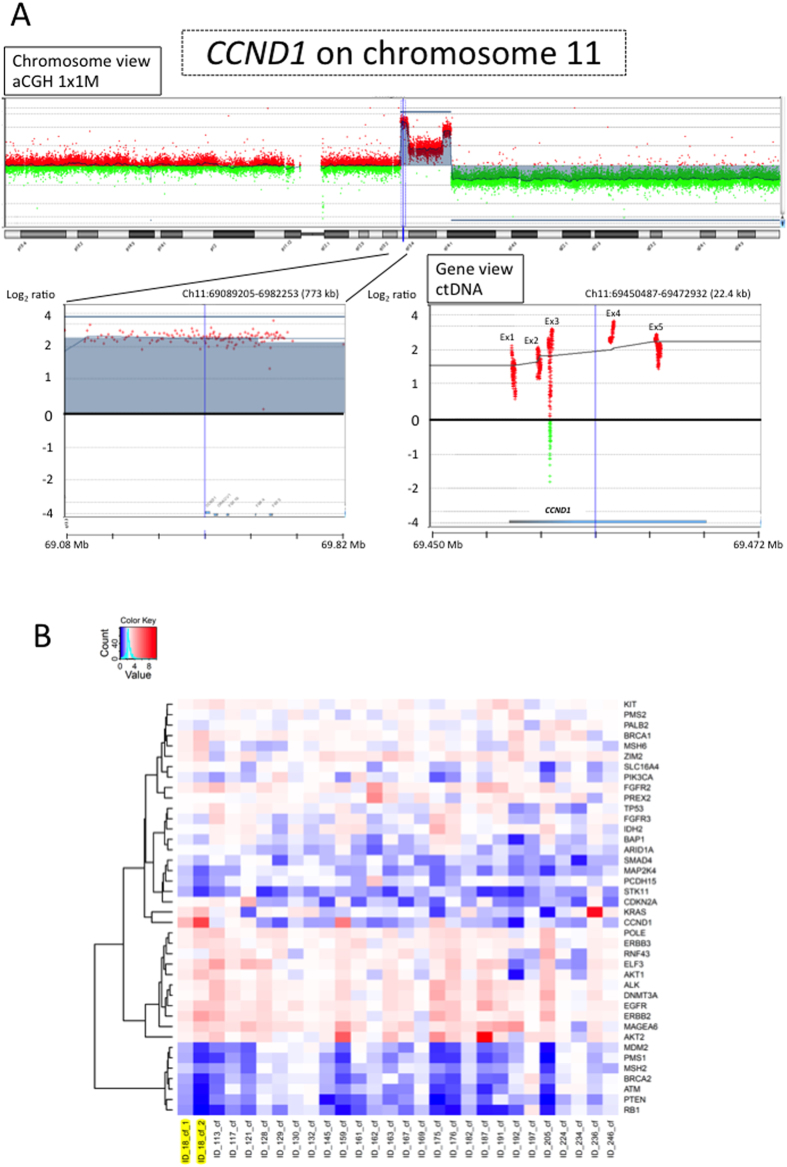
(**A**) Copy number alterations of the *CCND1* gene based on aCGH data of the primary cancer (*left*) and the targeted sequencing data of circulating tumor DNA (ctDNA) (*right*). In Patient ID-18, *CCND1* amplification was observed in both the aCGH data of the primary cancer and the targeted sequencing data of ctDNA. In the right figure, the plots indicate the adjusted copy number alterations at each genomic position based on the targeted sequencing data of ctDNA. Ex, Exon. (**B**) Copy number alterations in ctDNA. Copy number alterations were estimated based on targeted sequencing data. The copy number estimates were adjusted for purity of ctDNA in plasma cfDNA. In Patient ID-18 (highlighted in *yellow*), the copy number alterations were also analyzed by aCGH using frozen samples of the primary cancer and matched normal tissues. *CCND1* and *ERBB2* amplifications were observed in ctDNA of the patient, as identified in the aCGH analysis of gDNA generated from the primary tumor/normal tissues. The mutant *KRAS* allele frequency was used as a benchmark of the tumor purity on the Patient ID_18_cf_1 column. Since amplification of the *KRAS* gene was observed in aCGH of the primary cancer, the mutant *PBRM1* allele frequency was used as an alternative benchmark of the tumor purity on the Patient ID_18_cf_2 column. Patient ID-236 showed remarkable *KRAS* amplification, matching the results of droplet digital PCR (mutant allele frequencies of *KRAS*, 87.7%, Fig. 2D).

We also analyzed somatic copy number alterations based on the targeted sequencing data using our in-house algorithm. In one case (Patient ID-18), the results were compared with copy number alterations derived from array comparative genomic hybridization (aCGH) data obtained with frozen samples of the primary cancer and matched normal tissue (Supplementary Table S10). Among 60 genes selected for targeted sequencing, amplifications of the *CCND1* and *ERBB2* genes were detected in both ctDNA and the primary cancer (Fig. 4A and Supplementary Figure S7) in this patient. The copy number of each gene was adjusted for tumor purity of ctDNA in plasma cfDNA. Patients with low tumor variant allele frequencies (≤10%) in plasma cfDNA were excluded^27^. The somatic copy number alterations in 27 patients are shown in Fig. 4B.

## Discussion

We have analyzed plasma cfDNA in the largest number of PDAC patients (n = 259) ever reported using two different approaches, ddPCR and NGS. In general, these methods have advantages of sensitivity and comprehensiveness, respectively. We designed a strategy combining ddPCR (for prescreening) and NGS to utilize the respective advantages of each approach, in order to develop cost-effective and efficient methodology for analyzing ctDNA to identify mutations in PDAC patients.

In PDAC, a mutated *KRAS* gene is an attractive biomarker since *KRAS* mutation is observed in >90% PDAC and appears to occur during carcinogenesis^28^, so that almost all cancer cells within a tumor harbor *KRAS* mutations. In addition, mutation patterns of *KRAS* are limited in PDAC: four patterns (G12D, G12V, G12R and G13D) account for 90% of all mutations^21^,^22^. Therefore, we first attempted detection of mutated *KRAS* gene using the multiplex ddPCR assay. The running cost of the *KRAS* multiplex ddPCR assay is reasonable (approximately $50) with a quick turnaround time (15 hours from receiving the plasma sample to obtaining final data). The present study demonstrated that allele frequencies of mutant *KRAS* in plasma cfDNA varied from undetectable to 87.7%.

We have next attempted targeted deep sequencing using plasma cfDNA in PDAC patients who had either a high frequency of *KRAS* mutation detected by ddPCR or distant organ metastasis. By focusing on 60 genes, we were able to perform sequencing at higher coverage compared to the whole-exome sequencing of plasma cfDNA described in a previous report^29^. Sequencing coverage is a critical point with the NGS approach for plasma cfDNA, because the fraction of tumor DNA in plasma cfDNA is expected to be very low in general. However, increased PCR cycles in the library preparation from low input DNA would not achieve proportionally high sequence coverage because the PCR duplication rate is elevated in parallel. Therefore, we modified the target enrichment system (SureSelect-KAPA platform) without increasing PCR duplicates. Although exome data using cfDNA provide broader information on the cancer genome, our targeted deep sequencing platform could be more flexible, reliable and suitable for clinical application at a reasonable cost. Although targeted sequencing of ctDNA has recently been performed using commercial cancer panels, mostly by amplicon-based assay^18^,^30^,^31^,^32^, original gene panels specialized for cancer type should improve the detection efficiency of mutations in cfDNA. Most importantly, using our 60 PDAC gene panel, we found 17 mutations in potential therapeutic target genes in 14 of 48 (29.2%) patients from plasma cfDNA. The rate of finding genes with actionable somatic alterations in ctDNA was concordant with the targeted sequencing data recently reported by Sausen and colleagues^19^. These mutations in *ALK* (ALK inhibitors), *ATM* (DNA cross-linking drugs or poly (ADP-ribose) polymerase inhibitors), *DNMT3A* (DNMT inhibitors), *EGFR* (EGFR inhibitors), *KIT* (KIT inhibitors), *MAP2K4* (MEK inhibitors), and *PIK3CA* (PI3K/AKT/mTOR pathway inhibitors) are associated with FDA-approved therapies for oncologic indications or therapies in published prospective clinical studies, or are currently in human clinical trials with promising results^5^. For example, three of four mutations in *PIK3CA* are located in hot spots (p.H1047L, p.E545K and p.Q546K), according to the COSMIC database; they are known to be oncogenic and are *in vitro* therapy targets^25^,^26^. Because the mutations driving each tumor are unique, identifying the specific mutations in each patient’s cancer is critical for the development of a personalized treatment plan that takes advantage of the growing number of targeted therapies. These findings also imply that plasma cfDNA can be employed as an alternative to tissue biopsies for precision medicine to treat advanced PDAC.

We have also attempted the analysis of copy number alterations based on targeted sequencing data. In Patient ID-18, amplifications of the *CCND1* and *ERBB2* genes were suspected in ctDNA and the genes were also clearly amplified in the primary cancer by aCGH analysis (Fig. 4A and Supplementary Figure S7). Amplifications of *CCND1* and *ERBB2* genes are observed in PDAC^22^ and are potentially targetable^33^,^34^. Focusing on 27 patients with high percentages of ctDNA, high amplification could also be detected in our NGS platform. *ERBB2* amplification was seen in ctDNA in other cases (Fig. 4B), while not being validated in primary or metastatic tumor tissues. However, the accurate detection of copy number alterations, especially copy loss or homozygous deletion, is still challenging due to the low ctDNA fraction in plasma cfDNA in most PDAC patients (Supplementary Figure S8). Further improvements in capturing probes and analytical algorithms are needed to analyze copy number alterations of ctDNA.

There are several limitations to our study. First, the study design was retrospective, and blood was collected at a single time-point before treatment. In the future, prospective evaluation of *KRAS* mutations with multiple cfDNA sampling will be required to prove diagnostic potential for distant organ metastasis. Mutated *KRAS* genes could not be detected in the early stage of PDAC, so it would be premature to recommend the current protocol for screening to detect early-stage PDAC. Since PDAC is a hypovascular tumor, it is possible that DNA is scarcely released into the circulation in the early stages^9^. The sensitivity of the ddPCR assay we used is strongly dependent on the amount of template DNA fragment. In fact, previous studies^16^,^19^ confirm higher detectability of mutated *KRAS* in cfDNA in a larger volume of plasma (~5 mL) compared to our ddPCR analysis using cfDNA that corresponded to about 250 μL plasma. It may be possible to detect PDAC at an earlier stage with our liquid biopsy approach by using a larger volume of plasma.

In conclusion, we propose a novel approach for PDAC liquid biopsies: first analyze *KRAS* alleles using ddPCR as a benchmark and confirm the presence of sufficient ctDNA, and secondly, perform targeted sequencing of genes mutated frequently in PDAC and potential therapeutic target genes in patients with mutant *KRAS* alleles detected by ddPCR or patients with advanced PDAC with distant organ metastasis. We believe our approach could be cost-effective and applicable in the clinic. Finally, assessment of mutations and copy number alterations of plasma cfDNA may provide a new prognostic tool that would be helpful in deciding optimal therapeutic strategies for PDAC patients. Evaluation of ctDNA could be incorporated into future clinical trials as an alternative to primary or metastatic tumor biopsies for precision medicine in cancer cases.

## Methods

### Ethics statement

The experimental protocols were approved by the institutional review board at the National Cancer Center (2012-081). Written informed consent was obtained from all patients. The methods were carried out in accordance with the approved guidelines.

### Patients and plasma sample collection

The study involved patients with pancreatic ductal adenocarcinoma (PDAC) diagnosed between 2011 and 2014 at the National Cancer Center Hospital, Tokyo, Japan. Plasma and tissue samples were provided by the National Cancer Center Biobank with the approval of the National Cancer Center Biobank management committee. Tumors were diagnosed as PDAC from the histology of resected or biopsied materials. Patients were staged according to the classification of the Union for International Cancer Control (UICC) 7th edition. Clinicopathologic features of patients in this study are summarized in Supplementary Table S1. Peripheral venous blood samples were obtained before patients received any treatment, and were immediately processed to isolate plasma by centrifugation in EDTA tubes at 1,600 g for 10 minutes at 4 ^o^C. Plasma samples were aliquoted and stored at −80 ^o^C.

### Extraction and quantification of cfDNA

Before DNA extraction, plasma samples were centrifuged at 16,000 g for 10 minutes at 4^o^C in order to remove cell debris. Circulating cfDNA was extracted from 2 mL of plasma using the automated QIAsymphony extraction system and a QIAsymphony DSP Virus/Pathogen Midi kit (QIAGEN, Hilden, Germany) or using a QIAamp DNA Circulating Nucleic Acid kit (QIAGEN) according to the manufacturer’s instructions. Circulating cfDNA was eluted into 90 μL of elution buffer and stored at 4 ^o^C. Eluted cfDNA was quantified by SYBR Green I real-time PCR of human LINE-1 sequences^20^. PCR was performed in 20 μL reaction volume, containing 3 μL extracted cfDNA, 0.5 μM each of the forward primer (5′-TCACTCAAAGCCGCTCAACTAC-3′) and the reverse primer (5′-TCTGCCTTCATTTCGTTATGTACC-3′) and 1x iTaq SYBR Green Supermix (Bio-Rad, Hercules, CA). Amplification was carried out as follows: 2 minutes at 94 ^o^C and 35 cycles of 10 seconds at 94 ^o^C and 15 seconds at 58 ^o^C and 15 seconds at 70 ^o^C. A standard calibration curve was valid for 4-fold serial dilution of human genomic DNA (Promega, Madison, WI) up to 8 ng/reaction. Each sample was tested in triplicate.

### Detection of *KRAS* mutations by ddPCR

Multiplex ddPCR assays were carried out using the RainDrop digital PCR system (RainDance Technologies). We developed a *KRAS* 5-plex assay to detect the wild-type and the four most common mutations in PDAC: G12D, G12R, G12V and G13D (Fig. 2)^22^. The sequences of primers and probes are described in Supplementary Table S2. Samples for ddPCR was prepared by mixing 20 μL TaqMan Genotyping Master Mix (Thermo Fisher Scientific), 4 μL Droplet Stabilizer (RainDance Technologies), 0.5 μM each of forward and reverse primers, 6-FAM or VIC-conjugated TaqMan MGB probes (Thermo Fisher Scientific) and 11.2 μL of cfDNA with a median amount of 2.50 ng (range 0.61–114.36 ng) in a final reaction volume of 40 μL. Droplet generation, thermal cycling and signal detection were performed according to the manufacturer’s instructions. Data were analyzed using RainDrop Analyst software (RainDance Technologies). All results were reviewed manually, and then mutant signals without cluster formation were disregarded as false-positives. In each experiment, a positive control was prepared by mixing four types of *KRAS* mutant genomic DNA reference standards (Horizon Diagnostics, Cambridge, UK) (Fig. 2A).

### Targeted sequencing by the Illumina platform

Sixty genes were selected including recurrent mutated genes previously identified in PDAC^2^,^3^,^4^ and drug-targetable genes whose alterations were considered most likely to be clinically relevant (Supplementary Table S5). Plasma cfDNA samples, and germline DNA samples obtained from peripheral blood leukocytes were subjected to targeted capture sequencing using the SureSelect platform (Agilent Technologies). A sequence library was prepared using a combination of a KAPA Hyper Prep Kit (Kapa Biosystems) and the SureSelect Target Enrichment System (Agilent Technologies). End repair and A-tailing reactions were carried out in 50 μL reaction volumes containing 3 μL of End Repair & A-Tailing Enzyme Mix, 7 μL of End Repair & A-Tailing Buffer and 5–50 ng of input DNA. Reaction mixtures were incubated at 20 °C for 30 minutes and then 65 °C for 30 minutes in a thermal cycler without using a heated lid. Next, 50 μL aliquots of reaction products were added to adapter ligation reaction mixtures consisting of 10 μL DNA Ligase, 30 μL Ligation Buffer, 5 μL Agilent SureSelect Adapter Oligo Mix and 5 μL nuclease-free water. The 110-μL reaction mixtures were incubated at 20 °C for 15 minutes. Then 0.8x SPRI (Solid Phase Reversible Immobilization) cleanup was performed by adding 88 μL of AMPure XP (Beckman Coulter, Danvers, MA) reagent to the adapter-ligated DNA according to the manufacturer’s instructions. DNA was eluted in 20 μL of 10 mM Tris-HCl (pH 8.0). Post-capture library amplification reactions were carried out in 50 μL reaction volumes consisting of 25 μL 2x KAPA HiFi HotStart ReadyMix, 2.5 μL SureSelect Primer, 2.5 μL SureSelect ILM Indexing Pre capture PCR Reverse Primer and 20 μL adapter-ligated DNA. Reaction mixtures were subjected to a thermal cycling program as follows: 98 °C 45 seconds, 7–10 cycles of 98 °C 15 seconds, 65 °C 30 seconds, 72 °C 30 seconds, 1 cycle of 72 °C for 5 minutes. Cycle numbers were varied with the amount of input DNA (<10 ng, 10 cycles; 10–20 ng, 9 cycles; 20–30 ng, 8 cycles; >30 ng, 7 cycles). The amplified libraries were subjected to 1x SPRI cleanup by adding 50 μL of AMPure XP reagent to the PCR reaction products. Target capture and further library preparation processes were performed using the Agilent SureSelect Target Enrichment System according to the manufacturer’s instructions. Post-capture libraries were barcoded and pooled for sequencing. Sequencing was carried out on an Illumina HiSeq2000 (Illumina, Inc.).

### Detection of somatic mutations for the Illumina platform

Paired-end reads were aligned to the human reference genome (GRCh37) using the Burrows-Wheeler Aligner (BWA)^35^ for both cfDNA and matched germline DNA samples. Probable PCR duplications, for which paired-end reads aligned to the same genomic position, were removed, and pileup files were generated using SAMtools^36^ and a program developed in-house. To find somatic point mutations (single nucleotide variations (SNVs) and short indels), the following cutoff values were used for base selection: (i) a mapping quality score of at least 20; (ii) a base quality score of at least 10. Somatic mutations were selected using the following filtering conditions: (iii) the numbers of reads supporting a mutation for cfDNA were at least 8 and 12, for SNV and indel, respectively; (iv) the variant allele frequency of matched germline DNA was less than 0.03. Since sequence errors occur sequence-specifically, the read information of all germline DNA samples was grouped together in order to discriminate true positives from false positives accurately. Then the following filter was applied: (v) the ratio (NVAF/TVAF) of variant allele frequency of grouped germline DNA (NVAF) and cfDNA (TVAF) must be less than 0.1. Additionally the following filters were applied: (vi) when there were two or more somatic mutations (SNV and/or indel) within any 10 bp window, all of them with a TVAF of less than 0.2 were discarded; (vii) mutations with a root mean square mapping quality score of less than 40 for reads covering the mutation were discarded; (viii) mutations must be supported by both forward and reverse reads of more than 5% of all supported reads.

### Estimation of targeted sequencing with AmpliSeq technology

The mutations identified with the Illumina technology were evaluated using an alternative NGS technology based on amplicon deep sequencing. We selected appropriate primers to obtain optimal amplicons in mutations detected by the Illumina platform, and performed targeted sequencing with Ion AmpliSeq technology (Thermo Fisher Scientific). Circulating cfDNA (10 ng) was amplified 21 times using multiplex PCR. After the first round of PCR, sequence-specific primers were removed and the PCR products were phosphorylated. Ion-compatible adaptors were then ligated to the amplicons to prepare them for the second round of PCR amplification. After five cycles of PCR, the amplicons were purified and quantified for template preparation. Sequence template preparation was performed with OneTouch (Thermo Fisher Scientific). Template preparation was performed with an emulsion PCR. Templates were sequenced using the Ion PGM system (Thermo Fisher Scientific). Mutation analysis was performed using Torrent suite ver.4.2.1 and Torrent Variant Caller v4.2-18 (Thermo Fisher Scientific), and the in-house mutation-caller in the same manner as with the Illumina platform above, except for (i) not removing PCR duplications, (ii) not using the cutoff value for mapping quality score, (iii) with a cutoff value of base quality score of at least 15 and (iv) not applying additional filters.

### Validation of targeted sequencing by whole-exome sequencing for a primary cancer

Among 48 patients who were analyzed by targeted sequencing, snap-frozen samples were stored from one patient who underwent surgery (Patient ID-18). gDNA was extracted using QIAamp DNA Mini kit (Qiagen). Three micrograms of DNA per sample was sheared with a Covaris SS Ultrasonicator (Covaris, Woburn, MA). Exome capture was performed with an Agilent SureSelect Human All Exon Kit v5.0 (Agilent Technologies). Each sample was sequenced on an Illumina Hiseq2500 instrument using a read length of 2 × 100 bp. Paired-end reads were aligned to the human reference genome (GRCh37) using the Burrows-Wheeler Aligner (BWA)^35^ for both tumor and normal samples. Probable PCR duplications, for which paired-end reads aligned to the same genomic position, were removed, and pileup files were generated using SAMtools^36^ and a program developed in-house^37^. To find somatic SNVs and short indels, stringent confidence filtering conditions were applied. The details of our filtering conditions were as reported previously^38^.

### Validation of targeted sequencing by Sanger sequencing for the primary cancer

Validation of mutations was performed by Sanger sequencing using snap-frozen samples from PDAC patient ID-18. PCR amplification was carried out with 20 ng of gDNA using intronic primers flanking exons (Supplementary Table S11). PCR products were sequenced by use of a M13F primer (5′-GTAAAACGACGGCCAGT-3′) or M13R primer (5′-CAGGAAACAGCTATGACC-3′) incorporated into the forward and reverse primer of each primer pair, respectively. Sequencing data were analyzed with Sequencher 5.0.1 software (Gene Codes, Ann Arbor, MI). Mutation analysis, confirmation and determination of somatic status were carried out using the matched normal tissue from the same patient (Supplementary Figure S6).

### Detection of somatic copy number alterations in the primary cancer by aCGH

To analyze somatic copy number alterations in the primary cancer (Patient ID-18), gDNA from snap-frozen samples was hybridized to commercially available whole-genome tiling arrays consisting of one million oligonucleotide probes with an average spacing of 2.6 kb throughout the genome (SurePrint G3 Human CGH Microarray 1 × 1 M, Agilent Technologies). Data were analyzed using Genomics Workbench software (Agilent Technologies) according to the manufacturer’s instructions. We focused on the regions including 60 genes analyzed in targeted sequencing.

### Estimation of somatic copy number alterations of ctDNA with targeted sequencing data

Recent advances in DNA sequencing technology have allowed identification of somatic copy number alterations by NGS^39^,^40^. Copy number alterations should be adjusted for the tumor purity. In principal, this is generated by dividing tumor genomes by combined tumor and normal genomes. When analyzing copy number alterations of ctDNA based on targeted sequencing data, the mutant *KRAS* allele frequency can be used as a benchmark of the tumor purity, set as twice the mutant *KRAS* allele frequency. In cases with suspected *KRAS* amplification (i.e., ≥50% of the mutant *KRAS* allele frequency in plasma cfDNA: Patient ID-197 and ID-236), mutant *TP53* gene allele frequency was used instead. In cases with suspected wild-type *KRAS* and wild-type *TP53* (Patient ID-121 and ID-162), mutant *ARID1A* gene allele frequency was used as a benchmark of the tumor purity, since *ARID1A* was the gene with the highest allele frequency in both cases. Patients with a low tumor variant allele frequency in plasma cfDNA (≤10% of the mutant allele frequency of the benchmark gene) were excluded since it was considered likely to be difficult to estimate copy number alterations accurately^27^. The copy number alterations of ctDNA (ctCNA) in one region can be represented as follows: the genomic fraction estimates of ctDNA and non-tumor cfDNA in the target region/the genomic fraction estimates of cfDNA. Genomic coverage can be used as an alternative to genomic fraction estimates^41^. In addition, we adjusted the copy number alterations for tumor purity. Finally, our complete calculation formula was as follows: Adjusted copy number alteration of ctDNA = 1-(1-ctCNA)/tumor purity. We compared the copy number alterations based on targeted sequencing with those of aCGH in Patient ID-18. The mutant *PBRM1* gene allele frequency was used as a benchmark of the tumor purity, since the aCGH showed *KRAS* amplification in this case. In addition, since aCGH indicated that the wild-type allele of the *PBRM1* gene was lost, the tumor purity was adjusted using the following calculation formula: Tumor purity = 2x mutant *PBRM1* allele frequency/(mutant *PBRM1* allele frequency + 1). Among 60 targeted genes, amplifications of the *CCND1* and *ERBB2* genes were distinct in terms of copy number estimates based on both the aCGH data for the primary cancer and the targeted-sequencing data with plasma ctDNA (Fig. 4A and Supplementary Figure S7). Therefore, we compared the copy number estimates of the targeted genes based on the targeted-sequencing of ctDNA with those from aCGH data for the primary cancer. Somatic copy number alterations of each gene were estimated based on the relative sequence coverage from the coding regions of normal tissue/tumor pairs. The copy number of each “gene” was normalized by dividing the average sequence coverage of the coding region by the average sequence coverage of the entire coding regions. In Supplementary Figure S8, the plots indicate the average copy number estimates of each “gene”, based on aCGH (*x*-axis) and targeted-sequencing data (*y*-axis), adjusted for tumor purity. The findings suggest that estimation of somatic copy number alterations of ctDNA with targeted sequencing data might be useful if the usage condition is limited to amplification.

### Statistics

Correlations between *KRAS* mutational status and clinical data were statistically analyzed by χ^2^ test. Continuous variables were compared using the Student *t*-test. The principal outcome measure was length of survival as measured from the time of diagnosis. Patients alive at the time of follow-up were censored. The last follow-up point for patients still alive was April 2015. The Kaplan-Meier method was used to evaluate differences identified with the log-rank test. *P* < 0.05 was considered statistically significant. Variables that were found to be significant on univariate analysis were included in the multivariate analysis using Cox’s proportional hazards model. Statistical analyses were performed using JMP ver.11 (SAS Institute, Cary, NC).

## Additional Information

**How to cite this article**: Takai, E. *et al.* Clinical utility of circulating tumor DNA for molecular assessment in pancreatic cancer. *Sci. Rep.* **5**, 18425; doi: 10.1038/srep18425 (2015).

## Supplementary Material

Supplementary Information
