# Supplementary material for: Clinical utility of circulating tumor DNA for molecular assessment in pancreatic cancer
Source: Sci Rep. 2015 Dec 16;5:18425. doi: 10.1038/srep18425 (PMC4680882; doi:10.1038/srep18425)
Supplement: Supplementary Information [file srep18425-s1.pdf]

Supplementary Information for

Clinical utility of circulating tumor DNA for molecular assessment in pancreatic cancer

Erina Takai, Yasushi Totoki, Hiromi Nakamura, Chigusa Morizane, Satoshi Nara, Natsuko Hama, Masami Suzuki, Eisaku Furukawa, Mamoru Kato, Hideyuki Hayashi, Takashi Kohno, Hideki Ueno, Kazuaki Shimada, Takuji Okusaka, Hitoshi Nakagama, Tatsuhiro Shibata, Shinichi Yachida

This file includes:

- **Supplementary Table S1.**
- **Supplementary Table S2.**
- **Supplementary Table S3.**
- **Supplementary Table S4.**
- **Supplementary Table S5.**
- **Supplementary Table S6.**
- **Supplementary Table S7.**
- **Supplementary Table S8.**
- **Supplementary Table S9.**
- **Supplementary Table S10.**
- **Supplementary Table S11.**

- **Supplementary Figure S1.**
- **Supplementary Figure S2.**
- **Supplementary Figure S3.**
- **Supplementary Figure S4.**
- **Supplementary Figure S5.**

- **Supplementary Figure S6.**
- **Supplementary Figure S7.**
- **Supplementary Figure S8.**

Supplementary Table S1. Clinicopathological data.

	n	%
Gender		
Male	160	61.8
Female	99	38.2
Age (years)		
Mean	64.7	
Median	66	
Range	24-86	
Tumor location		
Head	119	45.9
Body/tail	140	54.1
Tumor size (mm)		
Mean	40.4	
Median	37	
Range	5-115	
T factor (UICC)		
T1	5	1.9
T2	6	2.3
T3	160	61.8
T4	81	31.3
Tx	7	2.7
N factor (UICC)		
N0	142	54.8
N1	115	44.4
Nx	2	0.8
M factor (UICC)		
M0	49	18.9

M1	208	80.3
Mx	2	0.8
UICC stage		
IA	3	1.2
IB	2	0.8
IIA	29	11.2
IIB	44	17.0
III	17	6.6
IV	163	62.9
Unknown	1	0.4

Supplementary Table S2. Sequences of primers and probes used for *KRAS* 5-plex droplet digital PCR.

Primers

	Sequence
Forward	5'-AGGCCTGCTGAAAATGACTGAATAT-3'
Reverse	5'-GCTGTATCGTCAAGGCACTCTT-3'

Probes

Target	Sequence	Reporter dye	Concentration
WT	5'-TTGGAGCTGGTGGCGT-3'	VIC	0.2 μM
G12D	5'-TGGAGCTGATGGCGT-3'	FAM	0.2 μM
G12R	5'-TTGGAGCTCGTGGCGT-3'	FAM	0.1 μM
G12V	5'-GAGCTGTTGGCGT-3'	FAM	0.3 μM
G13D	5'-CTGGTGACGTAGGCA-3'	FAM	0.3 μM
G13D	5'-CTGGTGACGTAGGCA-3'	VIC	0.2 μM

WT, wild type.

Supplementary Table S3. Possible clinical and pathologic risk factors for survival and detectability of mutant *KRAS* gene in plasma cfDNA (n = 259).

Variable	n	%	Overall survival	
			**Median (months)**	**Log-rank (*P* value)**
KRAS mutation in plasma cfDNA				< 0.0001
Negative	176	68.0	24	
Positive	83	32.0	9	
Tumor location				0.3203
Head	119	45.9	17	
Body/tail	140	54.1	17.6	
T factor (UICC)				0.0587
T1	5	1.9	34.8	
T2	6	2.3	-	
T3	160	61.8	19.6	
T4	81	31.3	12.6	
Tx	7	2.7		
N factor (UICC)				< 0.0001
N0	142	54.8	21.5	
N1	115	44.4	10.2	
Nx	2	0.8		
M factor (UICC)				< 0.0001
M0	49	18.9	34.8	
M1	208	80.3	14.7	
Mx	2	0.8		
UICC stage				< 0.0001
I-III	95	36.7	34.5	
IV	163	62.9	11.5	
NA	1	0.4		

Supplementary Table S4. Multivariate analysis of potential prognostic factors.

Variable	n	Hazard ratio	95% confidence limit	*P* value
KRAS mutation in plasma cfDNA				< 0.0001
Positive	83	3.037	2.023-4.559	
Negative	176	1		
N factor (UICC)				0.3175
N1	115	1.251	0.809-1.973	
N0	142	1		
M factor (UICC)				0.3609
M1	208	1.423	0.674-3.201	
M0	49	1		
UICC stage				0.0153
IV	163	2.054	1.147-3.771	
I-III	95	1		

Supplementary Table S5. Gene list for targeted sequencing.

	Gene	Approved or evaluated therapy	
1	***AKT1***	○	EGFR inhibitors
2	***AKT2***		
3	***ALK***	○	Tyrosine kinase inhibitors
4	***APC***		
5	***ARID1A***		
6	***ARID2***		
7	***ATM***	○	PARP inhibitors
8	***BAP1***		
9	***BRAF***	○	BRAF inhibitors
10	***BRCA1***	○	PARP inhibitors
11	***BRCA2***	○	PARP inhibitors
12	***CCND1***	○	CDK4 inhibitors
13	***CDK4***		
14	***CDKN2A***		
15	***DNMT3A***	○	DNMT inhibitors
16	***DPP6***		
17	***EGFR***	○	EGFR inhibitors
18	***ELF3***		
19	***EP300***		
20	***EPC1***		
21	***ERBB2***	○	ERBB2 inhibitors
22	***ERBB3***		
23	***ERBB4***		
24	***FGFR2***		
25	***FGFR3***	○	Tyrosine kinase inhibitor
26	***GNAS***		
27	***IDH1***		

28	***IDH2***		
29	***JAK1***		
30	***KIT***	○	Tyrosine kinase inhibitors
31	***KRAS***		
32	***MAGEA6***		
33	***MAP2K4***	○	MEK inhibitors
34	***MDM2***		
35	***MEN1***		
36	***MLH1***		
37	***MLL3***		
38	***MSH2***		
39	***MSH6***		
40	***NALCN***		
41	***PALB2***	○	Mitomycin C
42	***PBRM1***		
43	***PCDH15***		
44	***PIK3CA***	○	ERBB2 inhibitors, mTOR inhibitors, EGFR inhibitors, MEK Inhibitors
45	***PMS1***		
46	***PMS2***		
47	***POLE***		
48	***PREX2***		
49	***PRSS1***		
50	***PTEN***	○	BRAF inhibitors, ERBB2 inhibitors
51	***RB1***		
52	***RNF43***		
53	***SF3B1***		
54	***SLC16A4***		
55	***SMAD4***		
56	***STK11***		

57	***TGFBR2***
58	***TP53***
59	***UPF1***
60	***ZIM2***

Supplementary Table S6. Mutations detected by targeted deep sequencing (Illumina platform).

Pt. ID	Gene	Chr	Position	Mutation type	Mutation	Strand
18	***ARID1A***	1	27023909	in-frame_deletion	GCTGCGGCGGCGGCA>-	+
18	***PBRM1***	3	52696241	nonsense	G>A	-
18	***KRAS***	12	25398284	missense	C>T	-
49	***ARID1A***	1	27094370	frameshift_insertion	->T	+
49	***CDKN2A***	9	21971059	missense	G>C	-
49	***KRAS***	12	25398284	missense	C>A	-
49	***ARID2***	12	46230535	frameshift_deletion	G>-	+
49	***TP53***	17	7578395	missense	G>A	-
49	***SMAD4***	18	48573586	in-frame_deletion	TAA>-	+
110	***ARID1A***	1	27057916	nonsense	C>T	+
110	***KRAS***	12	25398284	missense	C>T	-
110	***POLE***	12	133220119	missense	G>T	-
110	***TP53***	17	7577498	splice_site	C>T	-
113	***ARID1A***	1	27023163	frameshift_deletion	GCGGC>-	+
113	***KRAS***	12	25398284	missense	C>A	-
113	***TP53***	17	7577120	missense	C>T	-
113	***SMAD4***	18	48603007	splice_site	G>T	+
117	***DNMT3A***	2	25458682	frameshift_insertion	->T	-
117	***PREX2***	8	68965427	nonsense	G>T	+
117	***CDKN2A***	9	21971142	nonsense	G>T	-
117	***KRAS***	12	25398281	missense	C>T	-
117	***TP53***	17	7578216	in-frame_deletion	AGTGTTTCTGTC>-	-
120	***KRAS***	12	25398284	missense	C>T	-
121	***ARID1A***	1	27057937	frameshift_insertion	->C	+
121	***PIK3CA***	3	178952085	missense	A>T	+

121	***MLL3***	7	151945340	frameshift_deletion	TC>-	-
121	***ARID2***	12	46231364	nonsense	G>T	+
121	***ARID2***	12	46298746	nonsense	C>A	+
121	***MAP2K4***	17	11924255	missense	A>G	+
127	***MLL3***	7	151935826	missense	T>A	-
127	***KRAS***	12	25398284	missense	C>T	-
127	***TP53***	17	7578262	missense	C>G	-
127	***TP53***	17	7578406	missense	C>T	-
128	***KRAS***	12	25398284	missense	C>A	-
128	***TP53***	17	7579471	frameshift_insertion	->G	-
129	***CDKN2A***	9	21971085	frameshift_deletion	CAGGAAGCCCTCCCGGG>-	-
129	***CDKN2A***	9	21971101	missense	G>C	-
129	***KRAS***	12	25398284	missense	C>T	-
129	***TP53***	17	7578406	missense	C>T	-
129	***PEG3***	19	57326922	missense	C>T	-
130	***PBRM1***	3	52712607	missense	C>T	-
130	***PIK3CA***	3	178916618	missense	C>T	+
130	***KRAS***	12	25398284	missense	C>A	-
130	***TP53***	17	7578525	missense	G>C	-
132	***ARID1A***	1	27056286	nonsense	C>T	+
132	***CDKN2A***	9	21974697	frameshift_insertion	->A	-
132	***KRAS***	12	25398284	missense	C>A	-
132	***TP53***	17	7577547	missense	C>A	-
132	***UPF1***	19	18976179	missense	A>G	+
137	***PIK3CA***	3	178936091	missense	G>A	+
137	***KIT***	4	55561831	missense	C>T	+
137	***CDKN2A***	9	21971186	nonsense	G>A	-
137	***KRAS***	12	25398284	missense	C>T	-
137	***TP53***	17	7578211	missense	C>T	-
137	***SMAD4***	18	48591901	missense	A>G	+

138	***DNMT3A***	2	25462035	missense	G>A	-
138	***DNMT3A***	2	25467207	missense	C>G	-
138	***KRAS***	12	25398284	missense	C>T	-
145	***DPP6***	7	154684101	missense	C>T	+
145	***PCDH15***	10	55944937	missense	A>C	-
145	***KRAS***	12	25398284	missense	C>A	-
155	***KRAS***	12	25398284	missense	C>A	-
155	***TP53***	17	7577089	frameshift_insertion	->GC	-
155	***BAGE3***	21	11058231	missense	G>A	-
159	***ARID1A***	1	27097714	nonsense	T>G	+
159	***CDKN2A***	9	21971111	missense	G>A	-
159	***KRAS***	12	25398284	missense	C>T	-
159	***TP53***	17	7574030	frameshift_deletion	G>-	-
161	***KRAS***	12	25398284	missense	C>A	-
161	***TP53***	17	7577153	missense	C>A	-
161	***SMAD4***	18	48584542	nonsense	C>T	+
162	***ARID1A***	1	27106100	frameshift_insertion	->A	+
162	***ALK***	2	29917736	missense	C>T	-
163	***CDKN2A***	9	21970971	nonsense	G>C	-
163	***KRAS***	12	25398284	missense	C>T	-
163	***TP53***	17	7578271	missense	T>A	-
164	***PCDH15***	10	55582194	in-frame_deletion	AGGAGC>-	-
164	***PCDH15***	10	55582217	missense	A>G	-
164	***PCDH15***	10	55663049	missense	C>T	-
164	***KRAS***	12	25398284	missense	C>T	-
164	***TP53***	17	7578556	splice_site	T>C	-
165	***MLL3***	7	151878287	frameshift_deletion	GA>-	-
165	***DPP6***	7	154585896	missense	C>T	+
165	***KRAS***	12	25398284	missense	C>A	-

167	***MSH6***	2	48032088	missense	G>A	+
167	***KRAS***	12	25398284	missense	C>A	-
167	***TP53***	17	7578271	missense	T>C	-
169	***ARID1A***	1	27087373	frameshift_insertion	->C	+
169	***CDKN2A***	9	21971087	frameshift_deletion	G>-	-
169	***KRAS***	12	25398284	missense	C>T	-
169	***NALCN***	13	101910920	missense	C>A	-
169	***TP53***	17	7576897	nonsense	G>A	-
169	***MAP2K4***	17	12011204	missense	T>A	+
175	***KRAS***	12	25398284	missense	C>T	-
175	***TP53***	17	7577156	splice_site	C>A	-
176	***KRAS***	12	25398284	missense	C>A	-
176	***TP53***	17	7577121	missense	G>A	-
176	***PEG3***	19	57328317	missense	C>G	-
178	***EGFR***	7	55241722	missense	G>A	+
178	***KRAS***	12	25398284	missense	C>T	-
178	***TP53***	17	7578190	missense	T>C	-
182	***PBRM1***	3	52651408	missense	A>G	-
182	***KRAS***	12	25398285	missense	C>A	-
182	***PEG3***	19	57326752	missense	C>T	-
186	***DNMT3A***	2	25471089	frameshift_insertion	->C	-
186	***KRAS***	12	25398284	missense	C>T	-
186	***TP53***	17	7578212	nonsense	G>A	-
187	***PREX2***	8	69136861	missense	G>C	+
187	***KRAS***	12	25398284	missense	C>A	-
187	***SMAD4***	18	48593422	nonsense	T>A	+
191	***KRAS***	12	25398284	missense	C>A	-
192	***PREX2***	8	69032431	missense	G>T	+
192	***KRAS***	12	25398284	missense	C>A	-

192	***TP53***	17	7574001	frameshift_insertion	->T	-
197	***ARID1A***	1	27023151	frameshift_deletion	GCGGAGCCGGCAGCGG CGGCGGGCCCGGC>-	+
197	***DNMT3A***	2	25470909	frameshift_deletion	GTACTCTGGCTCGTCAT CGCCTGCTTTG>-	-
197	***CDKN2A***	9	21971111	missense	G>A	-
197	***KRAS***	12	25398284	missense	C>T	-
197	***TP53***	17	7579565	frameshift_deletion	T>-	-
197	***EP300***	22	41564765	nonsense	C>T	+
205	***ARID1A***	1	27106387	nonsense	G>T	+
205	***KRAS***	12	25398284	missense	C>T	-
205	***TP53***	17	7578263	nonsense	G>A	-
224	***ATM***	11	108201008	missense	C>A	+
224	***KRAS***	12	25398284	missense	C>T	-
233	***CDKN2A***	9	21971164	in-frame_deletion	AGC>-	-
233	***KRAS***	12	25398284	missense	C>T	-
233	***TP53***	17	7577100	missense	T>C	-
234	***KRAS***	12	25398284	missense	C>T	-
234	***TP53***	17	7577568	missense	C>T	-
234	***GNAS***	20	57484421	missense	G>A	+
236	***CDKN2A***	9	21970969	missense	A>T	-
236	***KRAS***	12	25398284	missense	C>T	-
236	***TP53***	17	7577115	missense	A>G	-
237	***ALK***	2	30143204	missense	C>T	-
237	***KRAS***	12	25398284	missense	C>T	-
237	***RB1***	13	48934204	frameshift_deletion	TA>-	+
237	***TP53***	17	7577138	missense	C>G	-
240	***KRAS***	12	25398285	missense	C>G	-
240	***SMAD4***	18	48573547	missense	T>A	+

241	***PIK3CA***	3	178936094	missense	C>A	+
241	***CDKN2A***	9	21971124	frameshift_deletion	GA>-	-
241	***KRAS***	12	25398284	missense	C>A	-
241	***TP53***	17	7576897	nonsense	G>A	-
241	***SMAD4***	18	48603032	nonsense	C>T	+
244	***ARID1A***	1	27059275	missense	A>G	+
244	***CDKN2A***	9	21974754	frameshift_deletion	C>-	-
244	***KRAS***	12	25398284	missense	C>T	-
244	***TP53***	17	7577498	splice_site	C>A	-
244	***SMAD4***	18	48573570	missense	G>T	+
246	***CDKN2A***	9	21971186	nonsense	G>A	-
246	***KRAS***	12	25398284	missense	C>T	-
246	***TP53***	17	7578406	missense	C>T	-
249	***DNMT3A***	2	25463586	missense	C>T	-
249	***PCDH15***	10	55582658	missense	C>T	-
249	***KRAS***	12	25398285	missense	C>G	-
249	***TP53***	17	7577580	missense	T>C	-
249	***GNAS***	20	57429152	missense	C>G	+
251	***KRAS***	12	25398284	missense	C>T	-
251	***NALCN***	13	101710365	missense	C>T	-
251	***GNAS***	20	57429619	missense	A>G	+
252	***KRAS***	12	25398284	missense	C>T	-
252	***TP53***	17	7577538	missense	C>T	-
253	***KRAS***	12	25398284	missense	C>T	-
258	***TP53***	17	7577498	splice_site	C>T	-

Chr, chromosome.

Supplementary Table S7. *KRAS* mutations in plasma cfDNA detected by ddPCR and targeted deep sequencing.

Pt. ID	Droplet digital PCR		Targeted deep sequencing	
	Pattern	VAF (%)	Pattern	VAF (%)
236	G12D	87.69	G12D	86.88
197	G12D	70.63	G12D	77.53
130	G12V	42.16	G12V	46.13
169	G12D	36.13	G12D	44.96
244	G12D	32.53	G12D	36.89
234	G12D	39.06	G12D	36.75
18	G12D	21.51	G12D	30.40
182	-	0.00	G12C	29.63
132	G12V	26.06	G12V	28.46
167	G12V	32.18	G12V	27.60
145	G12V	19.45	G12V	23.48
161	G12V	15.07	G12V	21.95
117	-	0.00	G13D	21.30
249	G12R	20.66	G12R	20.01
175	G12D	15.57	G12D	17.93
176	G12V	18.11	G12V	16.06
192	G12V	15.38	G12V	15.32
129	G12D	17.99	G12D	15.10
163	G12D	13.48	G12D	14.29
159	G12D	10.98	G12D	14.08
128	G12V	8.04	G12V	11.02
191	G12V	13.66	G12V	10.66
205	G12D	18.41	G12D	10.23
186	G12D	6.57	G12D	10.17
110	G12D	3.08	G12D	10.12

113	G12V	6.29	G12V	9.51
246	G12D	6.74	G12D	8.11
137	G12D	13.66	G12D	8.04
120	G12D	5.86	G12D	7.57
127	G12D	5.21	G12D	6.66
165	-	0.00	G12V	6.37
233	G12D	5.05	G12D	6.30
187	G12V	6.79	G12V	5.76
155	G12V	4.91	G12V	5.29
164	G12D	4.51	G12D	4.66
178	G12D	2.21	G12D	4.56
251	G12D	4.35	G12D	3.66
138	G12D	3.29	G12D	3.65
49	G12V	4.27	G12V	3.62
237	G12D	3.70	G12D	3.47
241	G12V	3.05	G12V	3.20
224	G12D	1.08	G12D	2.25
240	G12R	1.45	G12R	1.95
252	G12D	0.89	G12D	1.95
253	G12D	1.22	G12D	1.60
258	G12D	2.44	-	0.00

VAF, variant allele frequency.

Supplementary Table S8. Comparison of targeted sequencing data between SureSelect-HiSeq2000 and Ion PGM platforms.

SureSelect-HiSeq2000 platform					Ion PGM platform			
**Pt. ID**	**Gene**	**Mutation type**	**Mutation**	**VAF of tumor**	**Gene**	**Mutation calling**	**Mutation**	**VAF of tumor**
197	*ARID1A*	fs_del	GCGGAGCC GGCAGCGGCGG CGGGCCCGGC>-	12.8%	*ARID1A*	DEL	GCGGAGCC GGCAGCGGCGG CGGGCCCGGC>-	60.6%
197	*DNMT3A*	fs_del	GTACTCTGGC TCGTCATCGC CTGCTTTG>-	1.3%				
197	*CDKN2A*	missense	G>A	59.0%	*CDKN2A*	SNV	G>A	65.5%
197	*KRAS*	missense	C>T	77.5%	*KRAS*	SNV	C>T	72.2%
197	*TP53*	fs_del	T>-	53.5%	*TP53*	DEL	T>-	61.0%
197	*EP300*	nonsense	C>T	27.5%	*EP300*	SNV	C>T	24.7&
234	*KRAS*	missense	C>T	36.8%	*KRAS*	SNV	C>T	36.0%
234	*TP53*	missense	C>T	40.0%	*TP53*	SNV	C>T	36.8%
234	*GNAS*	missense	G>A	29.8%	*GNAS*	SNV	G>A	26.3%
236	*CDKN2A*	missense	A>T	37.5%	*CDKN2A*	SNV	A>T	35.4%
236	*KRAS*	missense	C>T	86.9%	*KRAS*	SNV	C>T	87.0%
236	*TP53*	missense	A>G	43.1%	*TP53*	SNV	A>G	41.0%
237	*ALK*	missense	C>T	0.7%				
237	*KRAS*	missense	C>T	3.5%	*KRAS*	SNV	C>T	2.4%
237	*RB1*	fs_del	TA>-	6.1%				
237	*TP53*	missense	C>G	2.9%	*TP53*	SNV	C>G	3.5%
244	*ARID1A*	missense	A>G	0.6%				
244	*CDKN2A*	fs_del	C>-	24.9%	*CDKN2A*	DEL	C>-	31.2%
244	*KRAS*	missense	C>T	36.9%	*KRAS*	SNV	C>T	29.9%
244	*SMAD4*	missense	G>T	37.2%	*SMAD4*	SNV	G>T	33.7%

249	*DNMT3A*	missense	C>T	1.7				
249	*PCDH15*	missense	C>T	5.7	*PCDH15*	SNV	C>T	3.2
249	*KRAS*	missense	C>G	20.0	*KRAS*	SNV	C>G	20.5
249	*TP53*	missense	T>C	9.7	*TP53*	SNV	T>C	4.9
249	*GNAS*	missense	C>G	0.7				

VAF, variant allele frequency; fs_del, frame-shift deletion; DEL, deletion; SNV, single nucleotide variant.

Supplementary Table S9. Whole-exome sequencing data for the primary carcinoma (Patient ID-18).

Chr	Position	Types of mutations	Reference > Tumor allele	Gene	Strand	Number of tumor read	Number of reference read
1	37980322	missense	G>A	*MEAF6*	-	251	22202
1	43636480	nonsense	G>A	*EBNA1BP2*	-	183	6498
1	59042522	missense	G>A	*TACSTD2*	-	332	12351
1	112043018	missense	C>T	*ADORA3*	-	351	13223
1	169497288	missense	C>G	*F5*	-	180	12122
1	176809349	missense	G>A	*PAPPA2*	+	196	25816
2	148653986	missense	C>T	*ACVR2A*	+	405	32160
2	171822495	deletion	ACGCTGC CTCCT>-	*GORASP2*	+	286	36719
2	228881134	missense	C>T	*SPHKAP*	-	112	8234
2	230654338	missense	A>G	*TRIP12*	-	70	6109
3	42738590	missense	C>T	*HHATL*	-	48	3825
3	52696241	nonsense	G>A	*PBRM1*	-	277	9702
3	160804350	missense	A>C	*B3GALNT1*	-	434	16001
4	80898822	missense	G>A	*ANTXR2*	-	251	17698
4	155156833	missense	C>T	*DCHS2*	-	238	20165
5	135610444	missense	C>T	*TRPC7*	-	166	23714
5	149213062	missense	G>A	*PPARGC1B*	+	86	6381
5	153144013	missense	G>A	*GRIA1*	+	68	3026
6	27925302	missense	G>A	*OR2B6*	+	258	29368
6	123759216	missense	T>C	*TRDN*	-	91	6202
6	145956553	missense	A>T	*EPM2A*	-	253	17087
6	148855023	missense	G>T	*SASH1*	+	105	1626
7	100361777	missense	G>A	*ZAN*	+	124	5585
8	41551545	nonsense	C>A	*ANK1*	-	309	5375

8	103326118	missense	T>C	*UBR5*	-	552	23339
10	128019058	missense	C>G	*ADAM12*	-	67	71165
11	2185528	missense	G>A	*TH*	-	109	3079
11	61643905	missense	C>T	*FADS3*	-	82	6372
11	62294295	missense	C>T	*AHNAK*	-	195	17012
11	77690080	missense	G>C	*INTS4*	-	799	10797
12	1250928	missense	G>A	*ERC1*	+	145	5381
12	25398284	missense	C>T	*KRAS*	-	308	24131
12	112708086	missense	T>C	*C12orf51*	-	269	12810
13	36909281	missense	C>A	*SPG20*	-	269	9990
15	76995328	nonsense	G>A	*SCAPER*	-	151	8099
16	23409381	missense	C>T	*COG7*	-	138	7488
16	31098143	missense	C>T	*PRSS53*	-	127	3140
17	65907491	missense	A>T	*BPTF*	+	265	8937
18	8378314	missense	G>C	*PTPRM*	+	134	29166
18	8378356	missense	G>A	*PTPRM*	+	123	23629
18	8378361	missense	G>T	*PTPRM*	+	113	22936
18	8378379	missense	G>C	*PTPRM*	+	101	20769
18	19751625	deletion	GCCGCGGCG GCAGCA>-	*GATA6*	+	140	9937
19	408036	missense	C>T	*C2CD4C*	-	105	8229
19	4538265	missense	T>C	*LRG1*	-	110	13605
19	15758046	missense	G>A	*CYP4F3*	+	230	13229
19	47491324	splice_site	G>A	*ARHGAP35*	+	147	5811
19	47969129	missense	C>T	*SLC8A2*	-	167	8803
19	48244540	missense	G>A	*EHD2*	+	260	9593
19	51165470	missense	G>A	*SHANK1*	-	282	9074
19	51361374	missense	C>T	*KLK3*	+	337	27370
20	1209128	nonsense	C>T	*RAD21L1*	+	146	8492
20	5283038	missense	C>T	*PROKR2*	-	436	19844

20	43926884	missense	C>T	*MATN4*	-	185	24956
20	45174465	missense	C>T	*OCSTAMP*	-	151	10962
20	61978115	missense	G>A	*CHRNA4*	-	151	1312
22	30067822	deletion	G>-	*NF2*	+	53	2297
22	40697321	missense	A>G	*TNRC6B*	+	167	3837
23	24742468	missense	C>T	*POLA1*	+	55	3656
23	70621558	missense	A>G	*TAF1*	+	138	15429
23	129765459	missense	C>T	*ENOX2*	-	39	1497

Chr, chromosome.

Supplementary Table S10. Somatic copy number alterations for the primary carcinoma (Patient ID-18). The regions including 60 genes analyzed in targeted sequencing were focused.

Chr	Cytoband	Start	Stop	Amplification (CN)	Deletion (CN)	Target genes in the region
2	p25.3-p21	17019	47699548		1.280	*DMNT3A,MSH2*
2	p21-p14	47702953	68290400	4.136		*MSH2,MSH6*
3	p26.3-p11.1	62075	90309067		1.307	*BAP1,MLH1, PBRM1,TGFBR2*
3	q25.2-q29	152245400	197840339	2.752		*PIK3CA*
9	p24.3-p13.1	204104	39072270		1.238	*CDKN2A*
10	p15.3-p11.1	136361	38548507	2.808		*EPC1*
10	q21.3-q26.3	67590660	135435714		1.243	*FGFR2,PTEN*
10	q11.21-q21.1	44911726	55990287		1.250	*PCDH15*
11	q13.3-q14.1	68899781	77716982	4.156		*CCND1*
11	q13.3	68899781	70285927	9.020		*CCND1*
12	p13.31-p11.23	6458010	27556793	5.513		*KRAS*
12	p12.1-p11.23	23920579	27556793	4.875		*KRAS*
12	p12.1-p11.23	24725807	26551852	5.446		*KRAS*
14	q11.2-q32.33	19376762	107287505	3.602		*AKT1, APC*
17	q12-q21.31	33993027	42703848	3.219		*BRCA1,ERBB2*
17	q12-q21.2	37601154	39450169	3.649		*ERBB2*
17	p13.3-p12	47546	15065565		1.218	*MAP2K4,TP53*
17	q21.32-q24.2	47335050	65585605		1.239	*RNF43*
18	q11.2-q22.1	22996131	65408769		1.223	*SMAD4*

Chr, chromosome; CN, copy number.

Supplementary Table S11. *KRAS* and *PBRM1* primers for Sanger sequencing.

**Gene**		**Sequence (5'-3')**
KRAS	Forward	M13F-CGATACACGTCTGCAGTCAAC
	Reverse	TGAAACCCAAGGTACATTTCAG
PBRM1	Forward	GGTCTTTGCTGAAACAGGTGC
	Reverse	M13R-CAGAGGGAATCACAAGCAGG

M13F: GTAAAACGACGGCCAGT

M13R: CAGGAAACAGCTATGACC

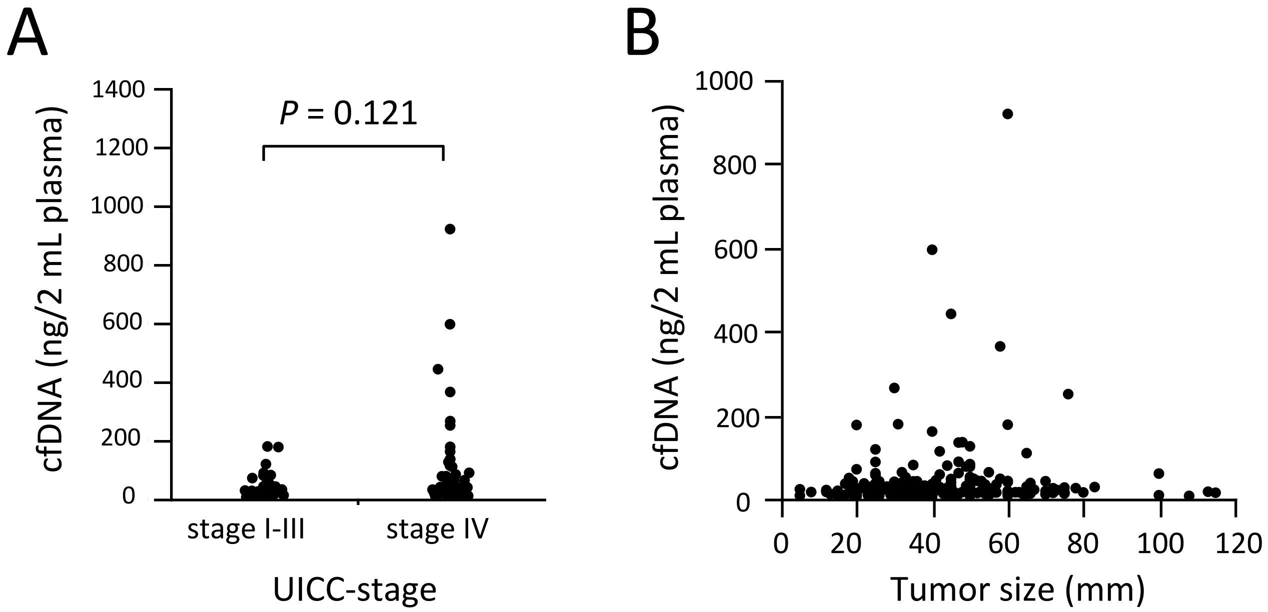

Supplementary Figure S1. Relationships between the amount of cfDNA and UICC-stages (*A*) and tumor size (*B*).

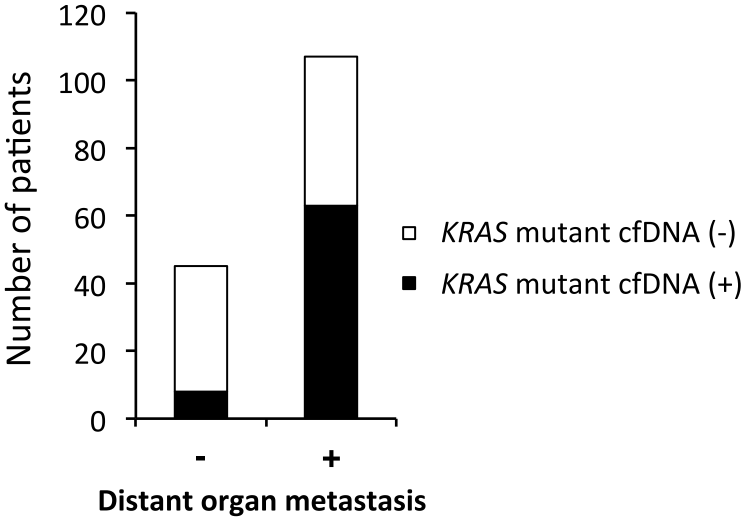

Supplementary Figure S2. Relationship between detectability of mutant *KRAS* in plasma cfDNA and distant organ metastasis at the time of diagnosis.

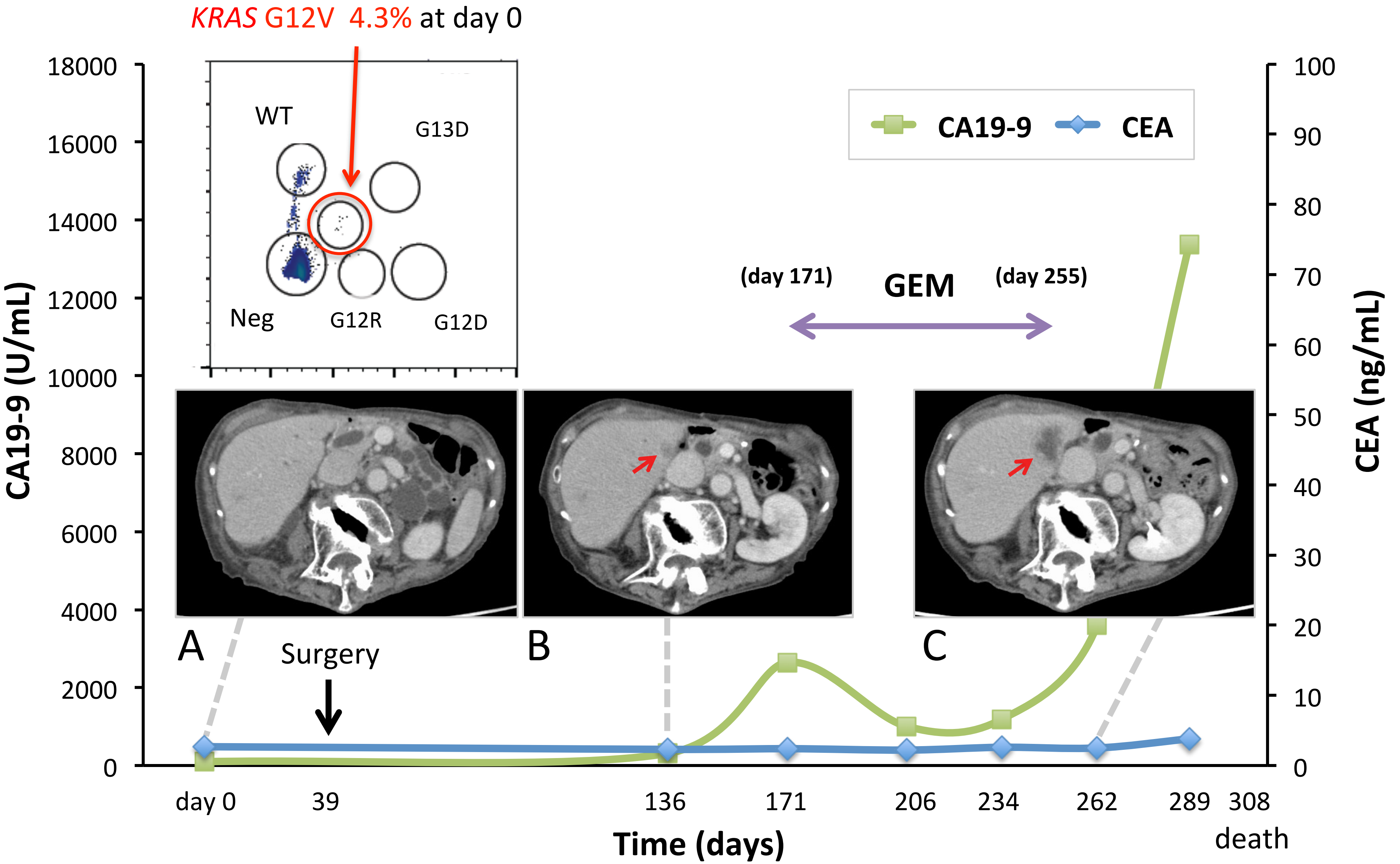

Supplementary Figure S3. Representative clinical course of a patient who had mutant *KRAS* alleles (G12V) preoperatively detected in plasma. CT images examined at the same time of plasma sampling (*A*), 3 months after the operation (*B*) and 2 months after the chemotherapy (*C*). Preoperative CT image (*A*) showed cystic lesions in the pancreas (main pancreatic tumor was detected as a 19 mm mass on other slices), while space-occupying lesion of the right lobe of the liver was not detected. After the operation, space-occupying lesion of the right lobe of the liver (*red arrow*) was detected (*B*) and increased in size (*C*) by CT scan. The patient died of the cancer 9 months after the operation. The CA19-9 and CEA levels were assessed during therapies. *Red arrows*, metastatic tumor in the liver. WT, wild-type; GEM, gemcitabine.

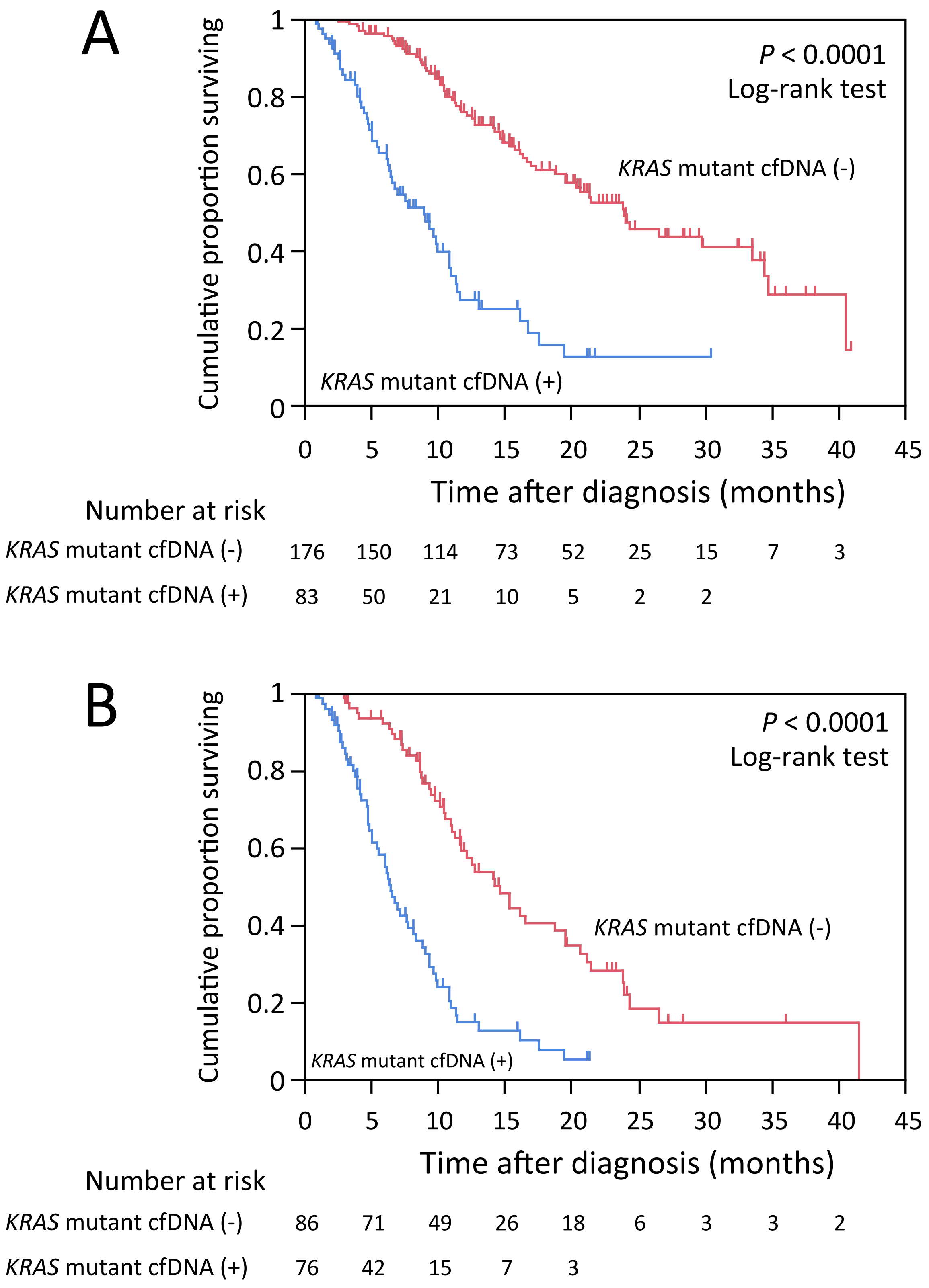

Supplementary Figure S4. Kaplan-Meier analysis of overall survival by detection of mutant *KRAS* in plasma cfDNA. (*A*) Patients with UICC-stages IA-IV cancer (n = 259). (*B*) Patients with UICC-stage IV cancer (n = 163).

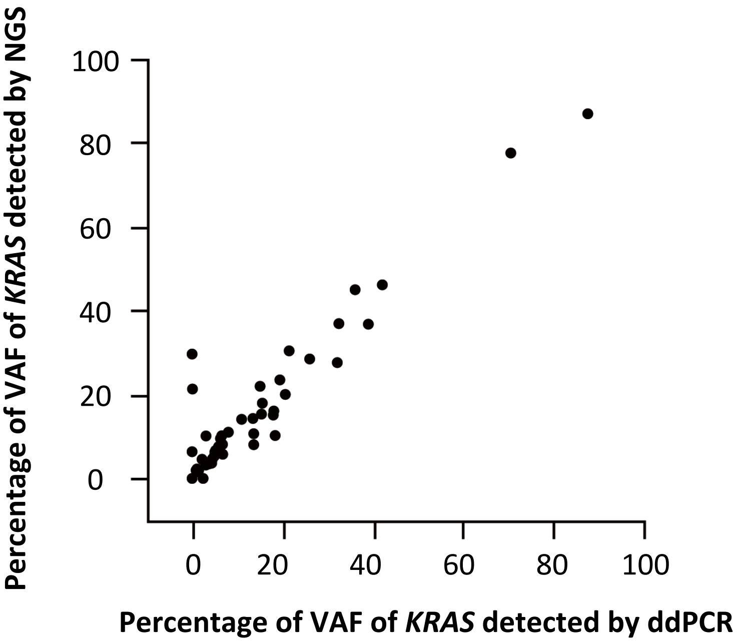

Supplementary Figure S5. Comparison between the percentage of variant allele frequency (VAF) of *KRAS* detected by ddPCR and that of VAF of *KRAS* detected by NGS, the Illumina platform.

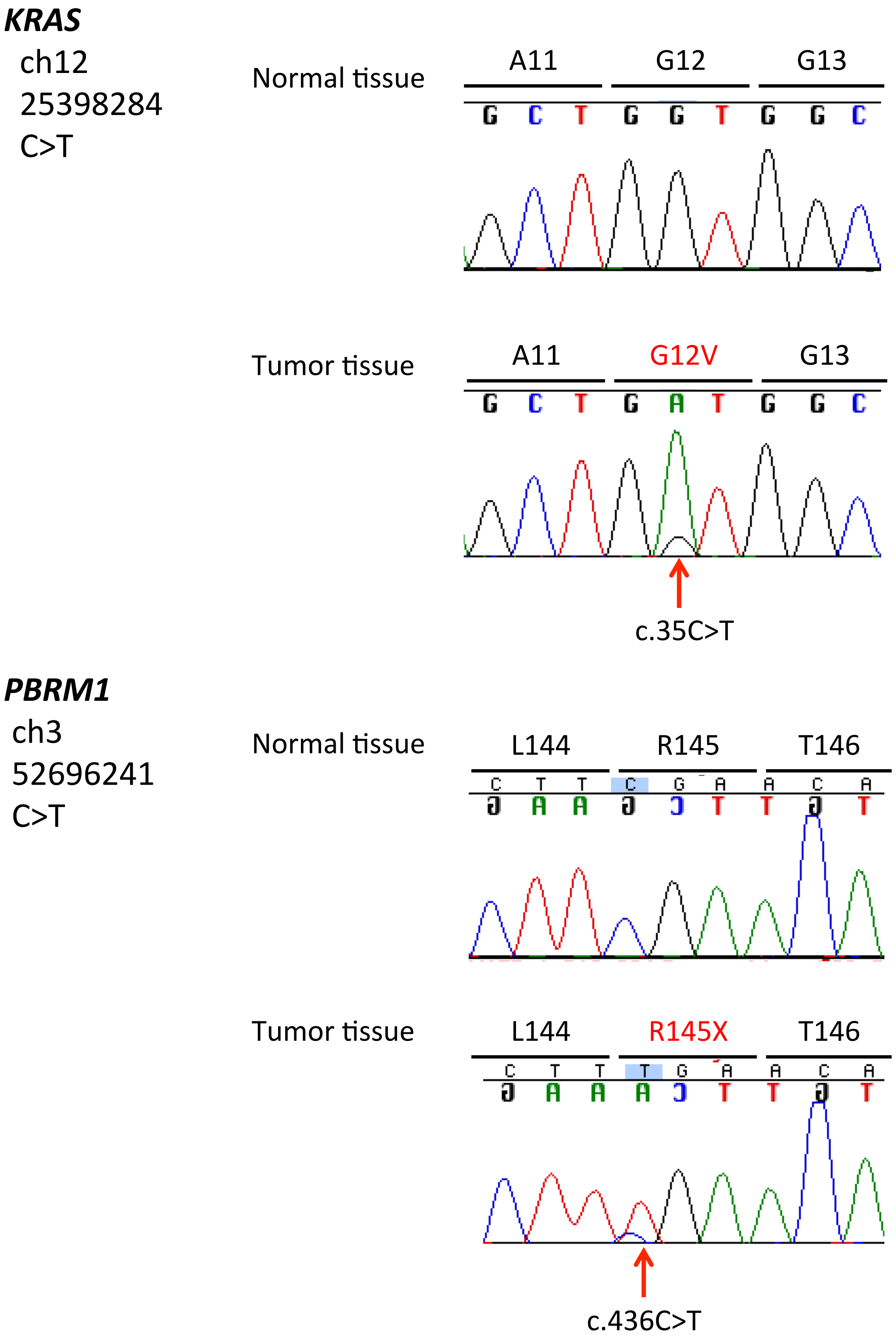

Supplementary Figure S6. Sanger sequencing analysis for *KRAS* and *PBRM1* in the primary cancer and the matched normal tissue (Patient ID-18). The same mutations as those were detected in plasma cfDNA were observed in the corresponding tumor tissue.

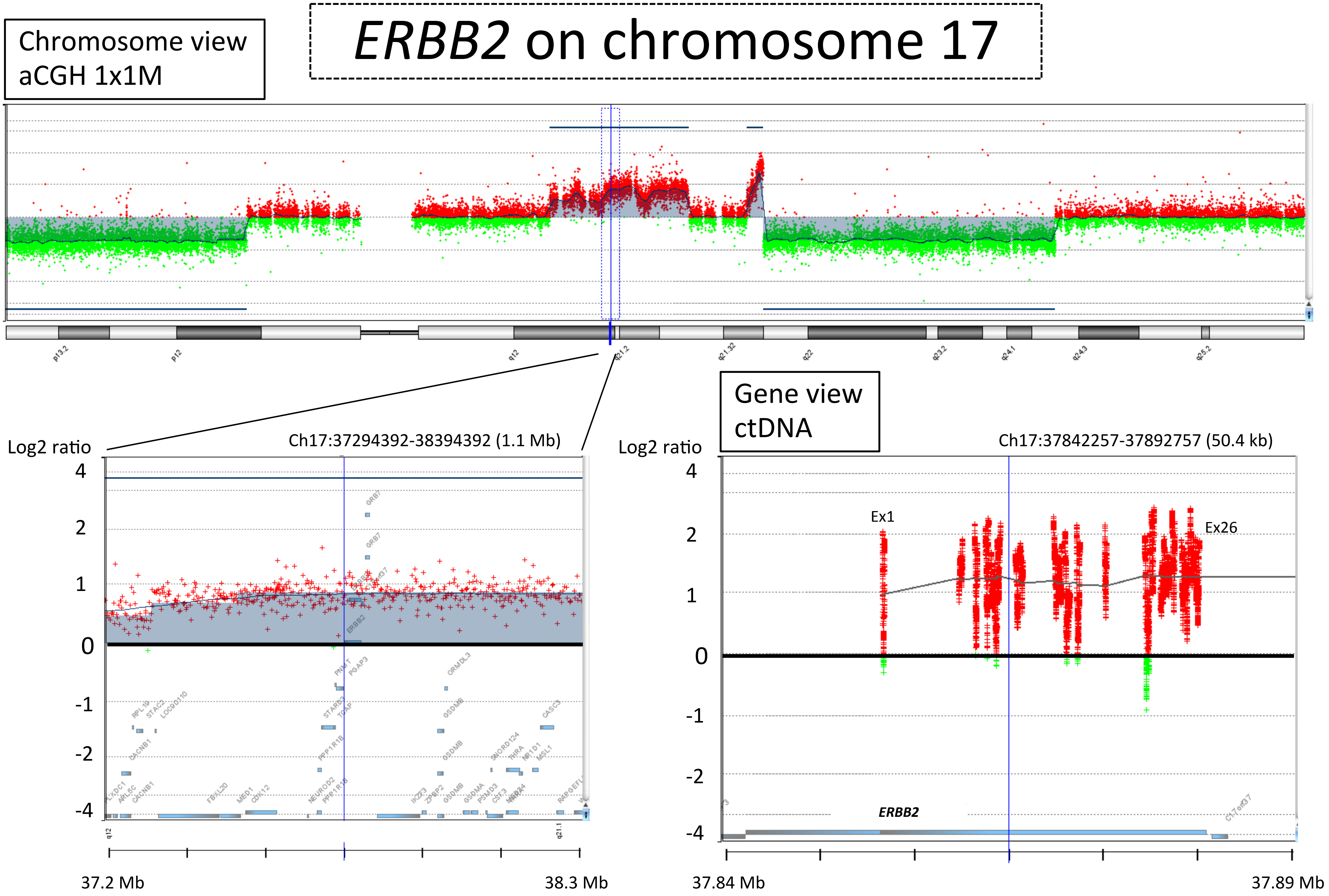

Supplementary Figure S7. Copy number alterations of the *ERBB2* gene based on aCGH data of the primary cancer (*left*) and the targeted sequencing data of ctDNA (*right*). Ex, Exon.

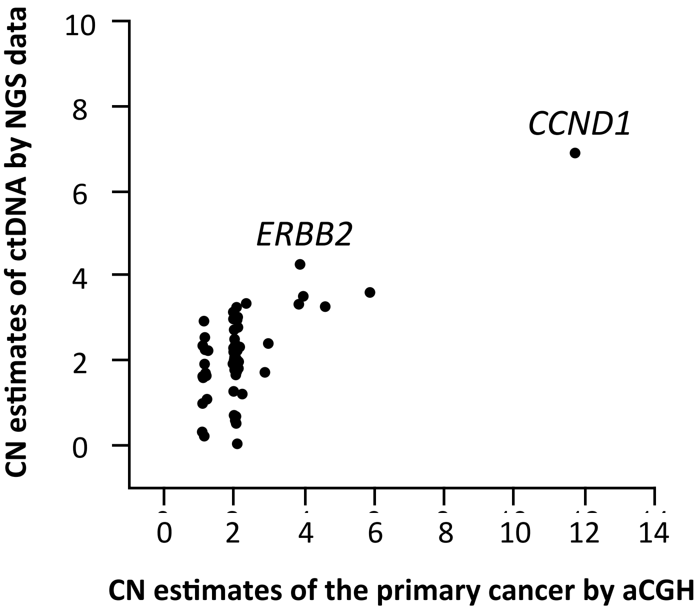

Supplementary Figure S8. Comparison of the copy number (CN) estimates of ctDNA based on the targeted sequencing data (*y* axis) with those based on aCGH data (*x* axis) for targeted 60 genes.
